# The Anti-Apoptotic Effects of Caspase Inhibitors in Propyl Gallate-Treated Lung Cancer Cells Are Related to Changes in Reactive Oxygen Species and Glutathione Levels

**DOI:** 10.3390/molecules27144587

**Published:** 2022-07-18

**Authors:** Woo hyun Park

**Affiliations:** Department of Physiology, Medical School, Research Institute for Endocrine Sciences, Jeonbuk National University, 20 Geonji-ro, Deokjin, Jeonju 54907, Jeollabuk, Korea; parkwh71@jbnu.ac.kr

**Keywords:** lung cancer, propyl gallate, cell death, caspase inhibitor, ROS, GSH

## Abstract

Propyl gallate [3,4,5-trihydroxybenzoic acid propyl ester; PG] exhibits an anti-growth effect in various cells. In this study, the anti-apoptotic effects of various caspase inhibitors were evaluated in PG-treated Calu-6 and A549 lung cancer cells in relation to reactive oxygen species (ROS) and glutathione (GSH) levels. Treatment with 800 μM PG inhibited the proliferation and induced the cell death of both Calu-6 and A549 cells at 24 h. Each inhibitor of pan-caspase, caspase-3, caspase-8, and caspase-9 reduced the number of dead and sub-G1 cells in both PG-treated cells at 24 h. PG increased ROS levels, including O_2_^∙−^, in both lung cancer cell lines at 24 h. Generally, caspase inhibitors appeared to decrease ROS levels in PG-treated lung cancer cells at 24 h and somewhat reduced O_2_^∙−^ levels. PG augmented the number of GSH-depleted Calu-6 and A549 cells at 24 h. Caspase inhibitors did not affect the level of GSH depletion in PG-treated A549 cells but differently and partially altered the depletion level in PG-treated Calu-6 cells. In conclusion, PG exhibits an anti-proliferative effect in Calu-6 and A549 lung cancer cells and induced their cell death. PG-induced lung cancer death was accompanied by increases in ROS levels and GSH depletion. Therefore, the anti-apoptotic effects of caspase inhibitors were, at least in part, related to changes in ROS and GSH levels.

## 1. Introduction

Propyl gallate [3,4,5-trihydroxybenzoic acid propyl ester; PG] is a synthetic antioxidant that has been used for decades as an additive for administered food, food packing, make-ups, and pharmaceutical preparations [1,2]. Although PG supposedly has a low toxicity, it exerts various positive effects on tissue and cellular functions. Several studies demonstrate the beneficial effects of PG as an antioxidant [3,4,5] and as a chemopreventive agent [6]. In contrast, PG treatment also has a slight pro-oxidant effect [7,8]. In addition, PG mediates its cytotoxicity via the disruption of mitochondrial function in endothelial cells, testicular cells, and hepatocytes [9,10,11]. The antioxidative and cytoprotective effects of PG could be altered to pro-oxidative, cytotoxic, and genotoxic effects in the presence of Cu(II) [12]. Additionally, PG augmented the growth of human fibroblasts at a concentration of 10^−8^ M but lessened their growth at a concentration of 10^−6^ M or higher [13]. Importantly, the anti-growth effects of PG were demonstrated in various cell types, such as testicular [10], leukemia [14], hepatocellular carcinoma [15], HeLa cervical cancer [16,17], breast cancer [18] and endothelial cells [11,19].

Apoptosis is a cellular response to cytotoxic drugs. The mechanisms of apoptosis generally involve two main signaling pathways: the mitochondrial pathway and the cell death receptor pathway [20]. The caspase family is a family of cysteine aspartate proteases that are known to play a major role in apoptosis [21,22]. Members of this family are divided into initiator and executioner caspases. Initiator caspases include caspase-2, caspase-8 and caspase-9, and executioner caspases include caspase-3, caspase-6, and caspase-7 [22]. The executioner caspases exist in cells as inactive dimers and are activated by cleavage [22]. The main substrates for initiator caspases are executioner caspases. The initiation of apoptosis in the mitochondrial pathway is induced or accompanied by increasing the levels of pro-apoptotic proteins, including BAX, and decreasing the levels of anti-apoptotic proteins, including Bcl-2 protein, subsequently causing the loss of mitochondrial membrane potential (MMP; ∆Ψ_m_) [23]. The essential event in this mitochondrial pathway is the efflux of cytochrome *c* from the mitochondria to cytosol, where it successively forms an apoptosome complex with apoptotic protease-activating factor 1 and caspase-9, consequently leading to the activation of a major executioner caspase, caspase-3 [20,22,24]. The pathway related to cell death receptors is characterized by the association of cell death ligands to their death receptors, which stimulates the activities of caspase-8 and caspase-3 [22,25]. The activation of caspase-3 systematically disassembles cells by cleaving crucial proteins, particularly poly (ADP-ribose) polymerase. Therefore, an attractive strategy for cancer therapy is to target the manipulation of the apoptotic pathways.

Reactive oxygen species (ROS) include hydrogen peroxide (H_2_O_2_), hydroxyl radical (^∙^OH), and superoxide anion (O_2_^∙−^), which are very unstable and irritable oxygen moieties. ROS are intentionally produced by certain oxidases, such as nicotinamide adenine dinucleotide phosphate oxidase and xanthine oxidase and are constantly produced during oxidative phosphorylation in mitochondria in the form of O_2_^∙−^ [26]. Superoxide dismutase enzymes (SODs) convert O_2_^∙−^ to H_2_O_2_ [27], and then H_2_O_2_ is metabolized into O_2_ and H_2_O by catalase or glutathione (GSH) peroxidase [28]. Since different kinds and levels of ROS can be either functional or detrimental to tissue and cellular functions, the status of cellular redox is steadily regulated to avoid the impairment of tissues and cells. The precise apoptotic progressions induced by ROS are not entirely understood, and the apoptotic effects of certain pro-oxidants and antioxidants remain questionable.

Lung cancer is the leading cause of cancer-related deaths worldwide [29]. Recently, we reported that PG treatment inhibited the growth of lung cancer cells through apoptosis or GSH depletion [30,31]. In the present study, the anti-apoptotic effects of various pan-caspase, caspase-3, caspase-8, and caspase-9 inhibitors were investigated in PG-treated Calu-6 and A549 lung cancer cells in relation to changes in ROS and GSH levels.

## 2. Materials and Methods

### 2.1. Cell Culture

Human Calu-6 and A549 lung cancer cell lines were obtained from the American Type Culture Collection (Manassas, VA, USA). These cells were maintained in an incubator containing 5% CO_2_ at 37 °C. Cells were cultured in RPMI-1640 containing 10% fetal bovine serum (FBS; Sigma-Aldrich Co., St. Louis, MO, USA) and 1% penicillin–streptomycin (Gibco BRL, Grand Island, NY, USA). Cells were grown in 100 mm plastic cell culture dishes (BD Falcon. Franklin Lakes, NJ, USA) and harvested with trypsin-EDTA (Gibco BRL).

### 2.2. Reagents

PG was purchased from Sigma-Aldrich Co. It was dissolved in ethanol to generate a 200 mM stock solution. The inhibitors of pan-caspase (Z-VAD-FMK; benzyloxycarbonyl-Val-Ala-Asp-fluoromethylketon), caspase-3 (Z-DEVD-FMK; benzyloxycarbonyl-Asp-Glu-Val-Asp-fluoromethylketon), caspase-8 (Z-IETD-FMK; benzyloxycarbonyl-Ile-Glu-Thr-Asp-fluoromethylketon), and caspase-9 (Z-LEHD-FMK; benzyloxycarbonyl-Leu-Glu-His-Asp-fluoromethylketon) were obtained from R&D Systems, Inc. (Minneapolis, MN, USA) and dissolved in dimethyl sulfoxide (DMSO, Sigma-Aldrich Co., St. Louis, MO, USA) to generate 10 mM stock solutions. Cells were pretreated with caspase inhibitors for 1 h prior to treatment with PG. Ethanol (0.2%) and DMSO (0.3%) were used as a control vehicle. All stock solutions were wrapped in foil and kept at −20 °C.

### 2.3. Cell Proliferation Assay

The effects of PG and caspase inhibitors on the proliferation of lung cancer cells were determined using the trypan blue exclusion cell counting assay, as previously described [32]. In brief, 5 × 10^5^ cells/well were seeded in 24-well plates (Nunc, Roskilde, Denmark). They were pretreated with each caspase inhibitor (15 μM) for 1 h and then treated with 800 μM PG for 24 h. The cells were collected by trypsin digestion for trypan blue exclusion cell counting. For all experimental conditions, three replicates were used, and the experiment was performed at least twice.

### 2.4. Cell Growth Inhibition Assay

The effects of PG and caspase inhibitors on the growth of lung cancer cells were determined using 3-(4,5-dimethylthiazol-2-yl)-2,5-diphenyltetrazolium bromide (MTT, Sigma-Aldrich Co., St. Louis, MO, USA) assays. Briefly, 5 × 10^4^ cells were seeded into 96-well microtiter plates (Nunc). They were pretreated with each caspase inhibitor (15 μM) for 1 h and then treated with 800 μM PG for 24 h. Twenty μL of MTT solution (2 mg/mL in phosphate-buffered saline (PBS)) was added to each well. The plates were incubated for 4 h at 37 °C. The medium was removed via pipetting, and 100–200 μL of DMSO was added to each well to solubilize the formazan crystals. The optical density was measured at 570 nm with a microplate reader (Synergy™ 2, BioTekR Instruments Inc., Winooski, VT, USA). For all experimental conditions, three replicates were used, and the experiment was performed at least twice.

### 2.5. Cell Cycle and Sub-G1 Cell Analysis

Cell cycle and sub-G1 distributions in cells were determined using propidium iodide (PI, Ex/Em = 488 nm/617 nm; Sigma-Aldrich Co., St. Louis, MO, USA) staining, as previously described [33,34]. Briefly, 1 × 10^6^ cells in 60 mm culture dishes (BD Falcon) were pretreated with each caspase inhibitor (15 μM) for 1 h and then treated with 800 μM PG for 24 h. Cells were washed with PBS and then incubated with 10 μg/mL PI together with RNase (Sigma-Aldrich Co., St. Louis, MO, USA) at 37 °C for 30 min. The proportions of cells in different phases of the cell cycle or with sub-G1 DNA content were measured and analyzed with a FACStar flow cytometer (BD Sciences, Franklin Lakes, NJ, USA).

### 2.6. Detection of Apoptosis

Apoptosis was measured via annexin V-fluorescein isothiocyanate staining (FITC, Ex/Em = 488/519 nm; Life Technologies, Carlsbad, CA, USA), as previously described [32,34]. Briefly, 1 × 10^6^ cells in 60 mm culture dishes (BD Falcon) were pretreated with each caspase inhibitor (15 μM) for 1 h and then treated with 800 μM PG for 24 h. Cells were washed twice with cold PBS, resuspended in 200 μL of binding buffer (10 mM HEPES/NaOH pH 7.4, 140 mM NaCl, 2.5 mM CaCl_2_) at a concentration of 5 × 10^5^ cells/mL, and incubated at 37 °C for 30 min. Annexin V-FITC (2 μL) was added to the solution, and cells were analyzed with a FACStar flow cytometer (BD Sciences).

### 2.7. Measurement of Mitochondrial Membrane Potential (MMP; ΔΨ_m_)

MMP (ΔΨ_m_) was monitored using Rhodamine 123 (Ex/Em = 485/535 nm; Sigma-Aldrich Co., St. Louis, MO, USA), a fluorescent, cell-permeable, cationic dye that preferentially enters into mitochondria, which typically have highly negative MMP (∆Ψ_m_). Depolarization of MMP (∆Ψ_m_) results in the loss of Rhodamine 123 from the mitochondria and reduces the intracellular fluorescence intensity of this dye [34,35]. In brief, 1 × 10^6^ cells in 60 mm culture dishes (BD Falcon) were pretreated with each caspase inhibitor (15 μM) for 1 h and then treated with 800 μM PG for 24 h. Cells were washed twice with PBS and incubated with Rhodamine 123 (0.1 mg/mL) at a concentration of 5 × 10^5^ cells/mL at 37 °C for 30 min. Rhodamine 123 staining intensities were determined using a FACStar flow cytometer (BD Sciences). Rhodamine 123-negative cells indicated the loss of MMP (∆Ψ_m_) in lung cancer cells. MMP (ΔΨ_m_) levels in cells, except for Rhodamine 123-negative cells, were expressed as percentages compared with control cells.

### 2.8. Determination of Intracellular ROS and O_2_^∙−^ Levels

Intracellular ROS, such as H_2_O_2_, ^∙^OH, and ONOO^∙^, were measured using the oxidation-sensitive fluorescent probe dye 2^′^,7^′^-dichlorodihydrofluorescein diacetate (H_2_DCFDA, Ex/Em = 495 nm/529 nm; Invitrogen Molecular Probes, Eugene, OR), as previously described [16,34,36,37]. DCF is poorly sensitive to O_2_^∙−^. In contrast, dihydroethidium (DHE, Ex/Em = 518 nm/605 nm; Invitrogen Molecular Probes) is a fluorogenic probe that selectively interacts with O_2_^∙−^ among ROS [16,34]. In brief, 1 × 10^6^ cells in 60 mm culture dishes (BD Falcon) were pretreated with each caspase inhibitor (15 μM) for 1 h and then treated with 800 μM PG for 1 or 24 h. Cells were then washed in PBS and incubated with 20 µM H_2_DCFDA or DHE at 37 °C for 30 min. Mean DCF and DHE fluorescence values were detected using a FACStar flow cytometer (BD Sciences). Mean DCF and DHE levels were expressed as percentages compared with control cells.

### 2.9. Detection of Intracellular GSH Levels

Cellular GSH levels were evaluated using 5-chloromethylfluorescein diacetate (CMFDA, Ex/Em = 522 nm/595 nm; Invitrogen Molecular Probes), as previously described [16,34]. In brief, 1 × 10^6^ cells in 60 mm culture dishes (BD Falcon) were pretreated with each caspase inhibitor (15 μM) for 1 h and then treated with 800 μM PG for 1 or 24 h. Cells were then washed with PBS and incubated with 5 µM CMFDA at 37 °C for 30 min. The mean CMF fluorescence intensity was determined using a FACStar flow cytometer (BD Sciences). CMF-negative cells indicated the depletion of GSH content in lung cancer cells. Mean CMF levels in cells, except for CMF-negative (GSH-depleted) cells, were expressed as percentages compared with control cells.

### 2.10. Statistical Analysis

The results represent the mean of multiple independent experiments (mean ± SD). The data were analyzed using Instat software (GraphPad Prism 5.0, San Diego, CA, USA). The Student’s *t*-test or one-way analysis of variance with post hoc analysis using Tukey’s multiple comparison test was used for parametric data. The statistical significance was defined as *p* < 0.05.

## 3. Results

### 3.1. Effect of Caspase Inhibitors on Cell Proliferation in PG-Treated Lung Cancer Cells

**Figure 1:**
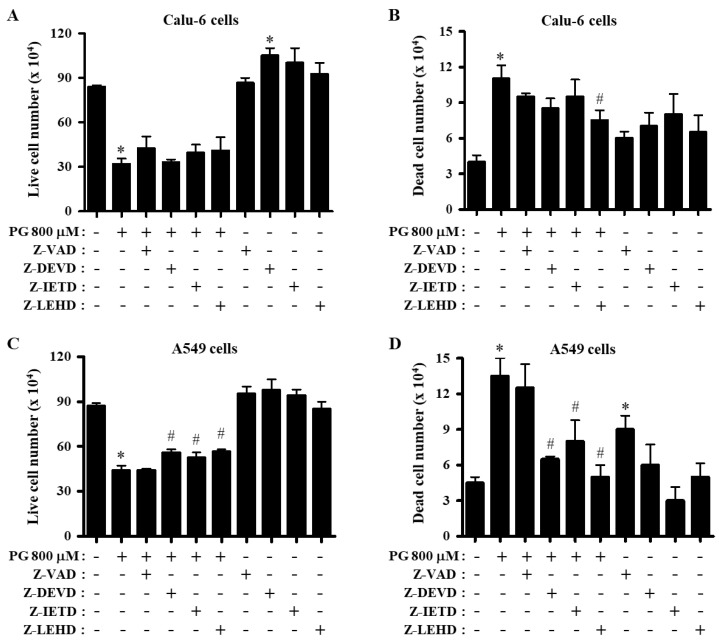
Effect of PG and caspase inhibitors on cell proliferation in Calu-6 and A549 lung cancer cells. Exponentially growing cells were incubated with 800 μM PG and/or each caspase inhibitor (15 μM) for 24 h. Live and dead cell numbers were assessed by the trypan blue exclusion cell counting assay. (**A**,**B**) Graphs show the numbers of Calu-6 live (**A**) and dead cells (**B).** (**C**,**D**) Graphs show the numbers of A549 live (**C**) and dead cells (**D**). * *p* < 0.05 compared with the PG-untreated control group. # *p* < 0.05 compared with cells treated with PG only. Data are presented as the mean ± SD. Z-VAD, pan-caspase inhibitor; Z-DEVD, caspase-3 inhibitor; Z-IETD, caspase-8 inhibitor; Z-LEHD, caspase-9 inhibitor.

The effects of PG and caspase inhibitors on the proliferation of Calu-6 and A549 lung cancer cells were examined by trypan blue cell counting. PG dose- and time- dependently inhibited the growths of Calu-6 and A549 cells, and an IC_50_ of PG was approximately 800 μM at 24 h, based on the MTT assays [30]. Based on previous experiments related to caspase inhibitors [32,37,38], cells were pretreated with inhibitors of pan-caspase (Z-VAD-FMK), caspase-3 (Z-DEVD-FMK), caspase-8 (Z-IETD-FMK), or caspase-9 (Z-LEHD-FMK) at a concentration of 15 μM before treatment with 800 μM PG, which was considered a suitable concentration to distinguish the number of live and dead cells in the presence or absence of each caspase inhibitor. Treatment with PG significantly decreased the proportion of Calu-6 live cells at 24 h (Figure 1A). Treatment with inhibitors of pan-caspase, caspase-8, or caspase-9 slightly increased the number of Calu-6 live cells treated with PG (Figure 1A). In addition, PG increased the number of Calu-6 dead cells, and all caspase inhibitors reduced the number of dead cells. In particular, the effect of the caspase-9 inhibitor was significant (Figure 1B). Generally, all caspase inhibitors appeared to increase the proliferation of control Calu-6 cells, including live and dead cells (Figure 1A,B). Furthermore, PG also decreased the number of A549 live cells, and caspase-3, caspase-8, and caspase-9 inhibitors attenuated the reduction in the number of A549 live cells induced by PG (Figure 1C). PG increased the number of A549 dead cells, and the inhibitors of caspase-3, caspase-8, and caspase-9 significantly reduced the number of A549 dead cells induced by PG (Figure 1D). In general, caspase inhibitors somewhat increased the proliferation of control A549 cells (Figure 1C,D).

### 3.2. Effects of Caspase Inhibitors on Growth and Cell Cycle Distributions in PG-Treated Lung Cancer Cells

**Figure 2:**
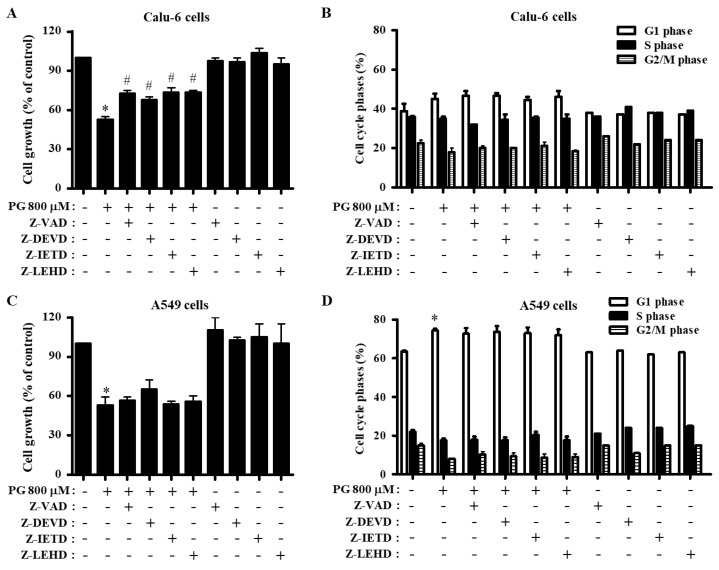
Effects of PG and caspase inhibitors on growth and cell cycle phase distributions in lung cancer cells. Exponentially growing cells were incubated with 800 μM PG and/or each caspase inhibitor (15 μM) for 24 h. Cell growth inhibition was evaluated by MTT assays. Cell cycle phase distributions were evaluated by DNA flow cytometry. (**A**,**B**) Graphs show cell growth (**A**) and cell cycle phase distributions in Calu-6 cells (**B**). (**C**,**D**) Graphs show cell growth (**C**) and cell cycle phase distributions in A549 cells (**D**). * *p* < 0.05 compared with the PG-untreated control group. # *p* < 0.05 compared with cells treated with PG only.

Treatment with 800 μM PG decreased the growth of Calu-6 cells by ~50% at 24 h, as measured by MTT assays (Figure 2A). All caspase inhibitors including pan-caspase inhibitor significantly prevented the inhibition of Calu-6 cells induced by PG (Figure 2A). Since changes in cell growth inhibition can be explained by an arrest during cell cycle progression, the distributions of cells in different phases of the cell cycle were observed in cells treated with PG and each caspase inhibitor after a 24 h incubation. The cell cycle progression consists of four phases: G1 phase, S phase, G2 phase, and M phase [39,40]. DNA flow cytometric analysis indicated that PG slightly induced G1 cell cycle arrest in Calu-6 cells (Figure 2B). None of the caspase inhibitors significantly affected the cell cycle distributions in PG-treated Calu-6 cells (Figure 2B). Furthermore, 800 μM PG reduced the growth of A549 cells by about 50% at 24 h (Figure 2C). Treatment with caspase-3 inhibitor slightly attenuated the growth reduction in A549 cells induced by PG (Figure 2C). In addition, PG significantly increased the proportion of A549 cells in G1 phase at 24 h, and none of the caspase inhibitors altered the cell cycle distributions of PG-treated A549 cells (Figure 2D).

### 3.3. Effects of Caspase Inhibitors on Cell Death and MMP (∆Ψ_m_) in PG-Treated Lung Cancer Cells

**Figure 3:**
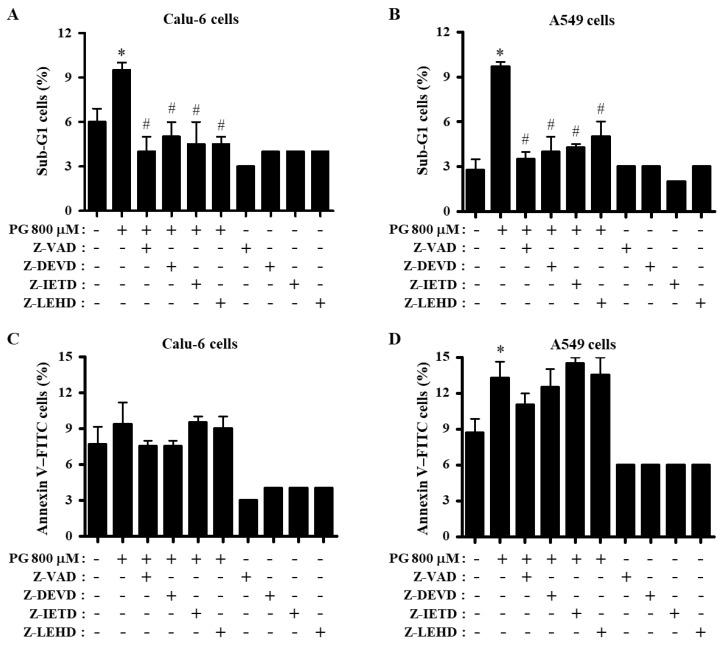
Effects of PG and caspase inhibitors on cell death in lung cancer cells. Exponentially growing cells were incubated with 800 μM PG and/or each caspase inhibitor (15 μM) for 24 h. Sub-G1 and annexin V-positive cells were measured with a FACStar flow cytometer. (**A**,**B**) Graphs show the proportions of sub-G1 Calu-6 cells (**A**) and A549 cells (**B**). (**C**,**D**) Graphs show the proportions of annexin V-positive Calu-6 cells (**C**) and A549 cells (**D**). * *p* < 0.05 compared with the PG-untreated control group. # *p* < 0.05 compared with cells treated with PG only.

Whether PG and caspase inhibitors affect cell death in Calu-6 and A549 cells was evaluated by determining the proportion of sub-G1 and annexin V-positive cells. As shown in Figure 3A,B, 800 μM PG increased the population of Calu-6 and A549 sub-G1 cells at 24 h. All caspase inhibitors significantly decreased the percentages of sub-G1 cells in both PG-treated Calu-6 and A549 cells (Figure 3A,B). Furthermore, 800 μM PG slightly increased the amount of annexin V-positive Calu-6 cells, and inhibitors of pan-caspase and caspase-3 decreased the number of annexin V-positive PG-treated Calu-6 cells to some extent (Figure 3C). In addition, PG significantly augmented the number of annexin V-positive A549 cells, and only the pan-caspase inhibitor (Z-VAD) slightly decreased the number of annexin V-positive PG-treated A549 cells (Figure 3D).

**Figure 4:**
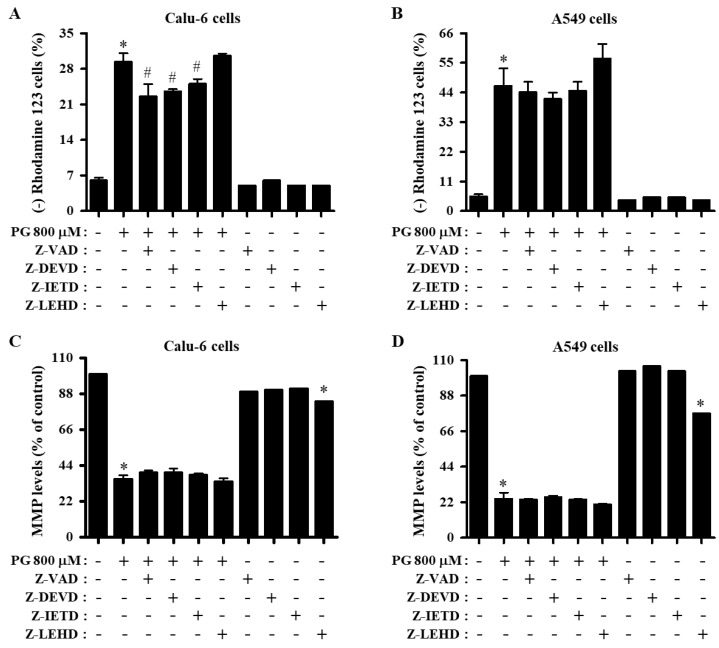
Effects of PG and caspase inhibitors on MMP (∆Ψ_m_) in lung cancer cells. Exponentially growing cells were incubated with 800 μM PG and/or each caspase inhibitor (15 μM) for 24 h. MMP (∆Ψ_m_) in lung cancer cells was measured using a FACStar flow cytometer. (**A,B**) Graphs show the proportions of Rhodamine 123-negative [MMP (∆Ψ_m_) loss] Calu-6 (**A**) and A549 cells (**B**). (**C**,**D**) Graphs indicate the proportions of MMP (∆Ψ_m_) levels in Calu-6 (**C**) and A549 cells (**D**). * *p* < 0.05 compared with the PG-untreated control group. # *p* < 0.05 compared with cells treated with PG only.

As apoptosis is closely correlated with the collapse of MMP (∆Ψ_m_), MMP (∆Ψ_m_) in cells treated with PG and caspase inhibitors was evaluated using Rhodamine 123 dye. After exposure to 800 μM PG for 24 h, the proportion of MMP (ΔΨ_m_) loss Calu-6 and A549 cells was approximately 30% and 45%, respectively (Figure 4A,B). While inhibitors for pan-caspase, caspase-3, and caspase-8 significantly decreased the loss of MMP (ΔΨ_m_) in PG-treated Calu-6 cells (Figure 4A), none of the caspase inhibitors reduced the loss of MMP (ΔΨ_m_) in PG-treated A549 cells (Figure 4B). With regard to the levels of MMP (ΔΨ_m_) in live lung cancer cells (except for MMP (ΔΨ_m_) loss cells), the levels of MMP (ΔΨ_m_) were approximately 35% and 22% in Calu-6 and A549 cells treated with 800 μM PG, respectively, compared with control cells (Figure 4C,D). None of the caspase inhibitors altered the levels of MMP (ΔΨ_m_) in either PG-treated Calu-6 or A549 cells (Figure 4C,D). All caspase inhibitors reduced the MMP (ΔΨ_m_) level in control Calu-6 cells, particularly the caspase-9 inhibitor, which significantly decreased the level in both lung cancer cell types (Figure 4C,D).

### 3.4. Effects of Caspase Inhibitors on ROS and O_2_^∙−^ Levels in PG-Treated Lung Cancer Cells

**Figure 5:**
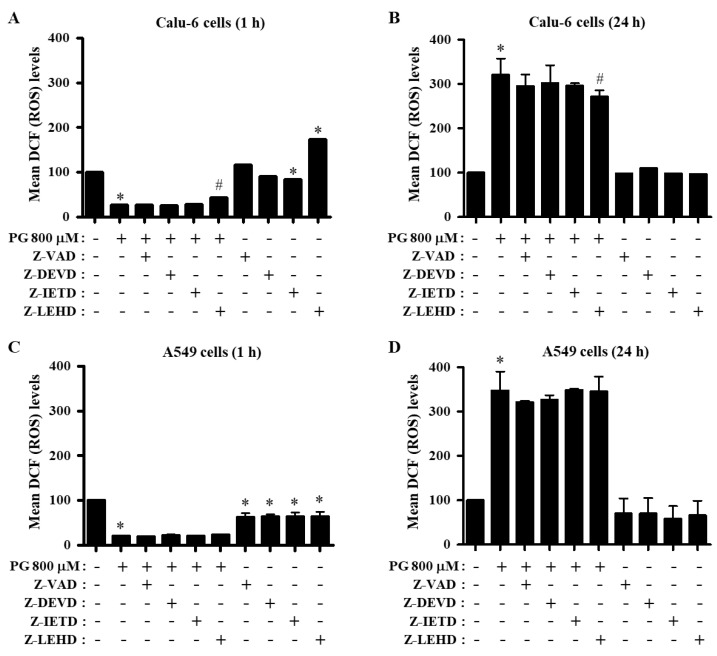
Effects of PG and caspase inhibitors on intracellular DCF (ROS) levels in lung cancer cells. Exponentially growing cells were incubated with 800 μM PG and/or each caspase inhibitor (15 μM) for 1 or 24 h. DCF (ROS) levels in lung cancer cells were measured using a FACStar flow cytometer. (**A**,**B**) Graphs indicate mean DCF (ROS) levels (%) in Calu-6 cells at 1 h (**A**) or 24 h (**B**) compared with control cells. (**C**,**D**) Graphs indicate mean DCF (ROS) levels (%) in A549 cells at 1 h (**C**) or 24 h (**D**) compared with control cells. * *p* < 0.05 compared with the PG-untreated control group. # *p* < 0.05 compared with cells treated with PG only.

To determine whether the levels of ROS in PG-treated lung cancer cells were changed by caspase inhibitors, intracellular ROS levels were assessed using H_2_DCFDA dye for nonspecific ROS and DHE dye for O_2_^∙−^. The ROS (DCF) level, including H_2_O_2_, was strongly decreased in PG-treated Calu-6 cells at 1 h, whereas the ROS level was significantly increased in these cells at 24 h (Figure 5A,B). Only the caspase-9 inhibitor attenuated the decreased ROS level in PG-treated Calu-6 cells at 1 h (Figure 5A). While inhibitors of pan-caspase and caspase-9 increased basal ROS levels in control Calu-6 cells at 1 h, caspase-3 and caspase-8 inhibitors decreased the basal level at this time (Figure 5A). All caspase inhibitors diminished the increased ROS level in PG-treated Calu-6 cells at 24 h, and the caspase-9 inhibitor showed a significant effect (Figure 5B). In addition, the ROS level was strongly decreased in PG-treated A549 cells at 1 h but significantly increased at 24 h (Figure 5C,D). None of the caspase inhibitors changed the ROS level in PG-treated A549 cells at either 1 or 24 h (Figure 5C,D). Interestingly, all caspase inhibitors decreased basal ROS levels in control A549 cells at both 1 and 24 h (Figure 5C,D).

**Figure 6:**
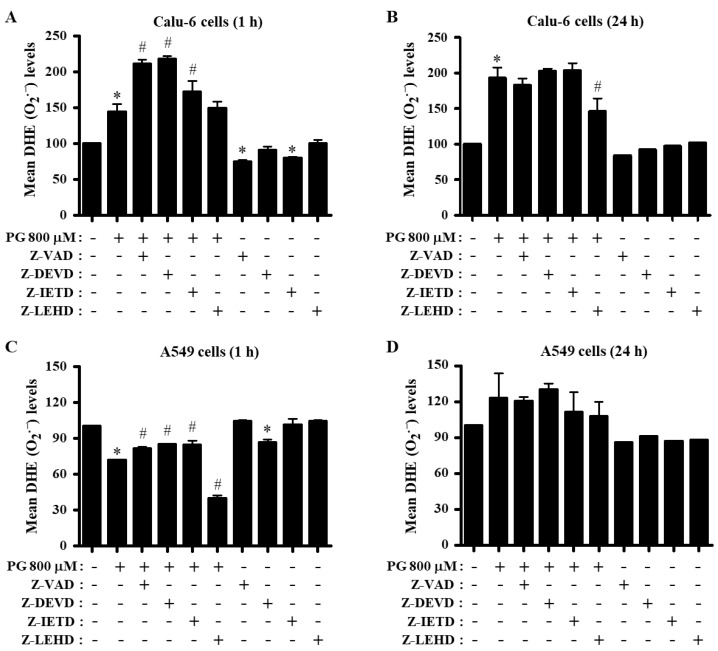
Effects of PG and caspase inhibitors on intracellular DHE (O_2_^∙−^) levels in lung cancer cells. Exponentially growing cells were incubated with 800 μM PG and/or each caspase inhibitor (15 μM) for 1 or 24 h. DHE (O_2_^∙−^) levels in lung cancer cells were measured using a FACStar flow cytometer. (**A**,**B**) Graphs indicate mean DHE (O_2_^∙−^) levels (%) in Calu-6 cells at 1 h (**A**) or 24 h (**B**) compared with control cells. (**C**,**D**) Graphs indicate mean DHE (O_2_^∙−^) levels (%) in A549 cells at 1 h (**C**) or 24 h (**D**) compared with control cells. * *p* < 0.05 compared with the PG-untreated control group. # *p* < 0.05 compared with cells treated with PG only.

The intracellular DHE (O_2_^∙−^) level was increased in Calu-6 cells treated with 800 μM PG at 1 and 24 h (Figure 6A,B). Inhibitors of pan-caspase, caspase-3, and caspase-8 enhanced the increased level of O_2_^∙−^ in PG-treated Calu-6 cells at 1 h (Figure 6A). Only the caspase-9 inhibitor significantly reduced the increased DHE (O_2_^∙−^) level induced by PG at 24 h (Figure 6B). The inhibitors of pan-caspase, caspase-3, and caspase-8 decreased basal O_2_^∙−^ levels in control Calu-6 cells at 1 and 24 h (Figure 6A,B). In A549 cells, PG significantly decreased the O_2_^∙−^ level at 1 h but increased the level at 24 h (Figure 6C,D). Inhibitors of pan-caspase, caspase-3, and caspase-8 attenuated the decreased O_2_^∙−^ level in PG-treated A549 cells at 1 h, whereas the caspase-9 inhibitor exaggerated the decreased level in these cells (Figure 6C). Only the caspase-3 inhibitor decreased the basal O_2_^∙−^ level in control A549 cells at 1 h (Figure 6C). None of the caspase inhibitors significantly altered the O_2_^∙−^ level in PG-treated A549 cells at 24 h, whereas all inhibitors reduced the basal O_2_^∙−^ level in control A549 cells at this time (Figure 6D).

### 3.5. Effects of Caspase Inhibitors on GSH Levels in PG-Treated Lung Cancer Cells

**Figure 7:**
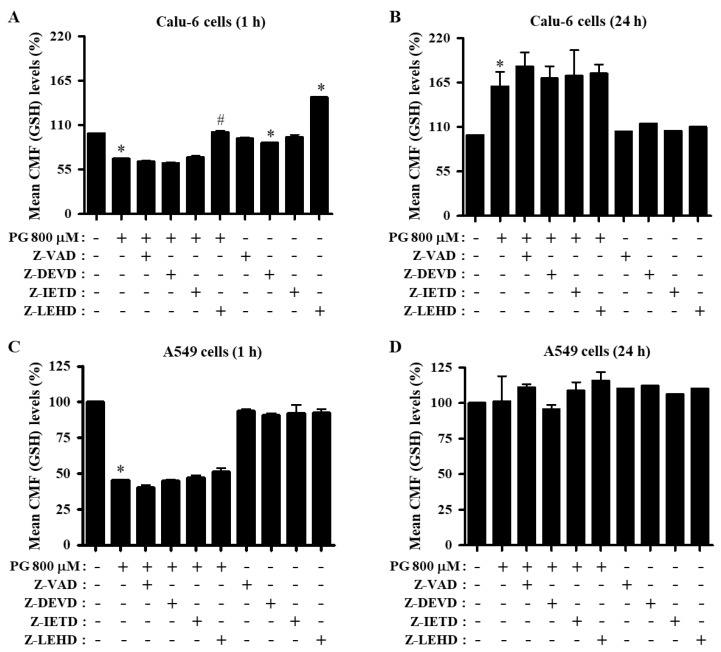
Effects of PG and caspase inhibitors on intracellular GSH levels in lung cancer cells. Exponentially growing cells were incubated with 800 μM PG and/or each caspase inhibitor (15 μM) for 1 or 24 h. Intracellular GSH levels in lung cancer cells were measured using a FACStar flow cytometer. (**A**,**B**) Graphs indicate mean CMF (GSH) levels (%) in Calu-6 cells at 1 h (**A**) or 24 h (**B**) compared with control cells. (**C**,**D**) Graphs indicate mean CMF (GSH) levels (%) in A549 cells at 1 h (**C**) or 24 h (**D**) compared with control cells. * *p* < 0.05 compared with the PG-untreated control group. # *p* < 0.05 compared with cells treated with PG only.

Changes in GSH levels were assessed in lung cancer cells treated with PG and caspase inhibitors using a CMF fluorescent dye. Treatment with 800 μM PG significantly decreased the GSH level in Calu-6 cells at 1 h but increased the level at 24 h (Figure 7A,B). Only the caspase-9 inhibitor prevented the decreased level of GSH in PG-treated Calu-6 cells at 1 h (Figure 7A). Inhibitors of pan-caspase, caspase-3, and caspase-8 downregulated the basal level of GSH in control Calu-6 cells at 1 h, whereas the caspase-9 inhibitor upregulated the basal level (Figure 7A). None of the caspase inhibitors significantly influenced GSH levels in PG-treated Calu-6 cells at 24 h (Figure 7B). In A549 cells, PG significantly diminished the GSH level at 1 h, whereas no effect was observed at 24 h (Figure 7C,D). None of the caspase inhibitors significantly altered GSH levels in PG-treated A549 cells at either 1 or 24 h (Figure 7C,D). Furthermore, all caspase inhibitors downregulated the basal level of GSH in control A549 cells at 1 h (Figure 7C).

**Figure 8:**
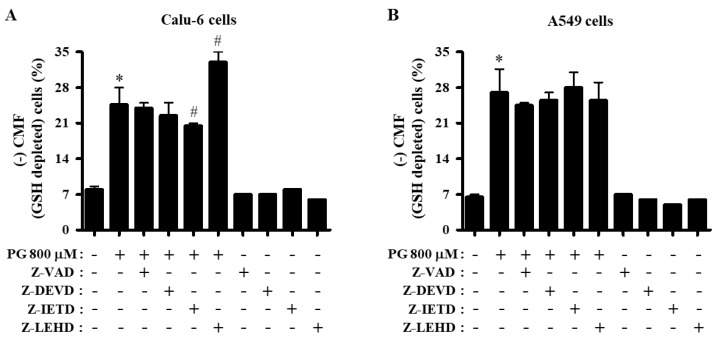
Effects of PG and caspase inhibitors on intracellular GSH depletion in lung cancer cells. Exponentially growing cells were incubated with 800 μM PG and/or each caspase inhibitor (15 μM) for 24 h. Intracellular GSH levels in lung cancer cells were measured using a FACStar flow cytometer. (**A**,**B**) Graphs show the percentage of negative CMF (GSH-depleted) Calu-6 cells (**A**) and A549 cells (**B**). * *p* < 0.05 compared with the PG-untreated control group. # *p* < 0.05 compared with cells treated with PG only.

When the number of GSH-depleted cells was measured in lung cancer cells at 24 h, 800 μM PG elevated the percentage of GSH-depleted Calu-6 and A549 cells compared with control cells (Figure 8A,B). The caspase-8 inhibitor significantly attenuated the depletion of GSH in PG-treated Calu-6 cells, whereas the caspase-9 inhibitor enhanced the depletion of GSH in these cells (Figure 8A). Furthermore, none of the caspase inhibitors significantly affected the depletion of GSH in PG-treated A549 cells (Figure 8B).

## 4. Discussion

Since PG inhibited the growth of Calu-6 and A549 lung cancer cell types through caspase-dependent apoptosis and GSH depletion [30], the present study focused on investigating the anti-apoptotic effects of various caspase inhibitors in PG-treated Calu-6 and A549 cells in relation to changes in ROS and GSH levels. Treatment with 800 μM PG significantly decreased the number of live Calu-6 and A549 cells at 24 h and increased the number of dead cells in both lung cancer cell lines. In addition, PG decreased the growth of Calu-6 and A549 cells by about 50% in the MTT assays. However, PG slightly increased cell death in both cancer cell lines by approximately 10%, as demonstrated by the analysis of sub-G1 and annexin V-positive cells. DNA flow cytometric analysis indicated that PG induced arrest at the G1 phase of the cell cycle in both lung cancer cell types. Therefore, G1 cell cycle arrest induced by PG is another feasible underlying mechanism for the inhibition of lung cancer cell growth. Furthermore, the number of sub-G1 cells in PG-treated lung cancer cells compared with control group cells was relatively higher than the number of annexin V-positive cells. Even 800 μM PG did not significantly increase the number of annexin V-positive Calu-6 cells, suggesting that PG induced necrosis and apoptosis. Collectively, PG inhibited the growth of lung cancer cells through the induction of G1 cell cycle arrest and cell death (apoptosis and necrosis). Furthermore, PG induced the loss of MMP (∆Ψ_m_) and reduced the MMP (∆Ψ_m_) level in Calu-6 and A549 cells. The proportion of MMP (∆Ψ_m_) loss cells in PG-treated Calu-6 cells was reduced compared with that in PG-treated A549 cells. The degree of MMP (∆Ψ_m_) loss cells in PG-treated lung cells was higher than that of annexin V-positive cells. These results suggest that PG initially affects the mitochondrial membrane in lung cancer cells, particularly in A549 cells, which initiates the next step of apoptosis.

Apoptosis occurs through mitochondrial (intrinsic) and cell death receptor (extrinsic) pathways [20]. All of the tested caspase inhibitors in this experiment significantly decreased the number of sub-G1 cells in PG-treated Calu-6 and A549 cells, and Z-VAD also reduced the number of annexin V-positive cells. In addition, most of the caspase inhibitors diminished the number of dead PG-treated cells and prevented the reduction in live cells. Moreover, inhibitors of pan-caspase, caspase-3, and caspase-8 decreased the percentage of MMP (∆Ψ_m_) loss in PG-treated Calu-6 and A549 cells. Therefore, the activation of caspase-3, caspase-8, and caspase-9 together appears to be necessary for the complete induction of apoptosis in PG-treated lung cancer cells. In fact, increases in caspase-3 and caspase-8 activities were observed in PG-treated Calu-6 cells [30]. These results suggest that PG-induced apoptosis in lung cancer cells involves both extrinsic and intrinsic pathways. However, some of the caspase inhibitors did not affect the proportion of annexin V-positive cells in PG-treated lung cancer cells. The caspase-8 inhibitor appeared to increase the number of annexin V-positive cells in PG-treated A549 cells. The caspase-9 inhibitor mildly enhanced MMP (∆Ψ_m_) loss in PG-treated Calu-6 and A549 cells and reduced the basal MMP (∆Ψ_m_) level in both control cells. Furthermore, some of the caspase inhibitors increased the number of dead cells in control Calu-6 and A549 cells. All caspase inhibitors also decreased the basal number of annexin V-positive control Calu-6 and A549 cells. The slight increase in the cell death of PG-treated or PG-untreated lung cancer cells by caspase inhibitors was perhaps caused by the induction of necrotic cell death following the inhibition of the apoptotic pathway by these inhibitors. Recently, it was reported that all of the inhibitors significantly prevented the PG-induced apoptosis in HeLa cervical cells [17] and some inhibitors somewhat exaggerated cell death in PG-treated calf pulmonary arterial endothelial (CPAE) cells [19]. All the tested caspase inhibitors enhanced the growth inhibition of PG-treated CPAE cells [19]. The current data demonstrated that caspase inhibitors attenuated growth inhibition in PG-treated Calu-6 cells but not A549 cells. Therefore, the different requirements for various caspases in PG-induced cell death and inhibition are probably dependent on cell types.

PG exhibits antioxidant [3,4,5,11,16] or pro-oxidant effects [7,8,15,16,37]. DCF (ROS) levels were decreased in both Calu-6 and A549 cells treated with 800 μM PG at 1 h, whereas the levels were usually increased in these cells at 24 h. In addition, DHE (O_2_^∙−^) levels in PG-treated Calu-6 and A549 cells were increased at 24 h. The current results generally indicate that the longer exposure of PG increased ROS levels, including O_2_^∙−^, in lung cancer cells (pro-oxidant effect), whereas its relatively shorter exposure decreased ROS levels (antioxidant effect). The significant prevention of PG-induced cell death by caspase inhibitors decreased DCF (ROS) levels in PG-treated lung cancer cells at 24 h and partly reduced O_2_^∙−^ levels. These results suggest that changes in ROS levels by PG and caspase inhibitors are, at least in part, related to the cell death of lung cancer cells. In contrast, the caspase-9 inhibitor did not affect the proportion of annexin V-positive or MMP (∆Ψ_m_) loss cells induced by PG, which resulted in a significant decrease in ROS levels, including O_2_^∙−^, in Calu-6 cells at 24 h. In addition, this inhibitor increased DCF (ROS) levels without affecting cell death in PG-treated or PG-untreated Calu-6 cells at 1 h. Inhibitors of pan-caspase, caspase-3, and caspase-8 also enhanced the increased level of O_2_^∙−^ in PG-treated Calu-6 cells at 1 h but had no effect on cell death. Furthermore, some of the caspase inhibitors decreased basal ROS levels, including O_2_^∙−^, in both control lung cancer cells. Explaining these results based on current knowledge is difficult. However, it is possible that the downregulated basal activities of caspases by their inhibitors improve the basal intact forms of antioxidant enzymes, such as SODs, catalase, and GSH peroxidase, to strongly scavenge basal ROS levels, including O_2_^∙−^, in lung cancer cells. The varying antioxidant and pro-oxidant effects of PG are perhaps due to the different basal activities of mitochondria and antioxidant enzymes in each cell type.

GSH is a non-protein thiol antioxidant that regulates cell proliferation, cell cycle progression, and cell death [41,42,43,44]. In addition, the intracellular GSH content is inversely related to the progression of cell death [42,43,44]. Likewise, PG increased the number of GSH-depleted Calu-6 and A549 cells at 24 h. The caspase-8 inhibitor significantly attenuated the depletion of GSH in PG-treated Calu-6 cells. In contrast, the caspase-9 inhibitor enhanced the depletion of GSH in PG-treated Calu-6 cells, and none of the caspase inhibitors affected the depletion of GSH in PG-treated A549 cells. Of note, the CMF (GSH) level in PG-treated Calu-6 cells was increased for 24 h. The increased GSH level may have occurred due to a decline in ROS levels, including O_2_^∙−^, induced by PG. At this point, cells beyond their capacity to resist oxidative stress are likely to die instantaneously. Interestingly, PG decreased CMF levels in both lung cancer cell lines at 1 h, especially in A549 cells. The reduced GSH level may have resulted from its urgent expenditure to diminish ROS (DCF) levels at this time. The caspase-9 inhibitor increased the level of GSH in PG-treated and PG-untreated Calu-6 cells at 1 h. In addition, pan-caspase, caspase-3, and caspase-8 inhibitors did not alter GSH levels decreased by PG, and these inhibitors downregulated the basal GSH level in control Calu-6 cells. None of the caspase inhibitors changed GSH levels in PG-treated A549 cells at 1 h, and all of them downregulated the basal level of GSH in control A549 cells. Moreover, none of the caspase inhibitors changed GSH levels in PG-treated Calu-6 and A549 cells at 24 h. Accordingly, these results suggest that PG and caspase inhibitors affect intracellular GSH levels differently depending on the cell type and incubation times, which are not closely related to lung cancer cell death.

In conclusion, PG exhibited anti-proliferative effects in Calu-6 and A549 lung cancer cells and induced their cell death through apoptosis and necrosis. PG-induced lung cancer death was accompanied by an increase in ROS and GSH depletion. The anti-apoptotic effects of caspase inhibitors on PG-induced lung cancer cell death were, at least in part, associated with changes in ROS and GSH levels. The present data provide useful information on the cell death effects of PG and caspase inhibitors in lung cancer cells with respect to changes in ROS and GSH levels.

## Data Availability

Data collected during the present study are available from the corresponding author upon reasonable request.

## Sample Availability

Samples of the compounds are not available from the authors.

## Abbreviations

PG: propyl gallate; ROS, reactive oxygen species; GSH, glutathione; SODs, superoxide dismutase enzymes; MMP (ΔΨ_m_), mitochondrial membrane potential; MTT, 3-(4,5-dimethylthiazol-2-yl)-2,5-diphenyltetrazolium bromide; FITC, fluorescein isothiocyanate; PBS, phosphate-buffered saline; Z-VAD-FMK, benzyloxycarbonyl-Val-Ala-Asp-fluoromethylketon; Z-DEVD-FMK, benzyloxycarbonyl-Asp-Glu-Val-Asp-fluoromethylketon; Z-IETD-FMK, benzyloxycarbonyl-Ile-Glu-Thr-Asp-fluoromethylketon; Z-LEHD-FMK, benzyloxycarbonyl-Leu-Glu-His-Asp-fluoromethylketon; H2DCFDA, 2^′^,7^′^-dichlorodihydrofluorescein diacetate; DHE, dihydroethidium; CMFDA, 5-chloromethylfluorescein diacetate.
